# Effectiveness of Organisational Strategies for Pressure Injury Prevention and Treatment in Acute Hospital Settings: A Systematic Review

**DOI:** 10.1111/jan.17090

**Published:** 2025-06-05

**Authors:** Jake McMahon, Elizabeth McInnes, Ching Shan Wan, Nicola Straiton, Louisa Lam, Jane Rodgers, Jessica Dickson, Paul Fulbrook

**Affiliations:** ^1^ School of Nursing, Midwifery and Paramedicine Australian Catholic University Fitzroy Victoria Australia; ^2^ St Vincent's Hospital Melbourne Fitzroy Victoria Australia; ^3^ National Health and Medical Research Council Centre of Research Excellence in Wiser Wound Care Griffith University Brisbane Queensland Australia; ^4^ Nursing Research Institute, St Vincent's Health Network Sydney, St Vincent's Hospital Melbourne Australian Catholic University Darlinghurst New South Wales Australia; ^5^ Respiratory Research @Alfred, School of Translational Medicine Monash University Melbourne Victoria Australia; ^6^ School of Nursing, Midwifery and Paramedicine Australian Catholic University North Sydney New South Wales Australia; ^7^ School of Public Health and Preventive Medicine Monash University Clayton Victoria Australia; ^8^ St Vincent's Health Network Sydney Darlinghurst New South Wales Australia; ^9^ RMIT University Library RMIT University Melbourne Victoria Australia; ^10^ School of Nursing, Midwifery and Paramedicine Australian Catholic University Brisbane Queensland Australia; ^11^ Nursing Research and Practice Development Centre, The Prince Charles Hospital Metro North Health Service District Brisbane Queensland Australia; ^12^ Faculty of Health Sciences, School of Therapeutic Sciences University of the Witwatersrand Johannesburg South Africa

**Keywords:** acute care, delivery of health care, hospitals, implementation strategies, pressure injury, prevention, systematic review

## Abstract

**Aim:**

To investigate the effects of organisational interventions on the incidence, healing and management of pressure injuries in adult patients in acute hospital settings.

**Design:**

Systematic review.

**Methods:**

The review included adult patients at risk of or with pre‐existing pressure injuries in acute hospital settings, excluding mental health units, emergency departments or operating theatres. Interventions employed in the included studies were categorised using the Cochrane Effective Practice and Organisation of Care taxonomy.

**Data Sources:**

Cochrane Central Register of Controlled Trials, Ovid MEDLINE, Ovid Embase, EBSCO CINAHL Complete and Web of Science Core Collection were searched from 01 January 2012 to 31 December 2023.

**Results:**

Of 8861 records identified, 7 prevention studies met the inclusion criteria. Six studies reported reductions in pressure injury incidence. Included studies employed various combinations of 14 organisational strategies to enhance practices. Educational interventions were utilised in six studies, including educational meetings, materials and outreach visits. Other common strategies included audit and feedback, communities of practice and continuous quality improvement. The interventions targeted patients and clinicians, primarily nurses, with some involving multidisciplinary teams. The focus was on enhancing healthcare practices through systematic approaches and stakeholder engagement.

**Conclusions:**

Organisational strategies targeting both patients and clinicians as part of an intervention bundle may enhance the prevention of pressure injuries in acute hospital settings. Further, high‐quality effectiveness–implementation hybrid trials are required to evaluate these strategies.

**Implications for the Profession and Patient Care:**

Organisational factors influence clinicians' ability to implement evidence‐based practices. The effectiveness of specific organisational strategies in acute settings is uncertain. Multiple organisational strategies targeting patients and clinicians may improve the implementability of a pressure injury prevention intervention.

**Reporting Method:**

This study adhered to PRISMA guidelines.

**Patient or Public Contribution:**

Neither patients nor the public were directly involved in this study.

**Summary**

- What does this paper contribute to the wider global clinical community?
  ○ Organisational strategies targeting both patients and clinicians for pressure injury prevention may be worthwhile, highlighting the need for high‐quality trials to evaluate their effectiveness.

## 1 Introduction

A pressure injury is localised damage to the skin and underlying tissue, usually over a bony prominence or related to a medical or other device: they are classified using four stages (I–IV) and three categories (suspected deep tissue injury, unstageable and mucous membrane; European Pressure Ulcer Advisory Panel et al. 2019). Longer hospital stays, limited mobility or conditions such as diabetes and spinal cord injuries increase the risk of developing pressure injuries, particularly in high‐pressure anatomical locations such as the sacrum, heels and hips (Afzali Borojeny et al. 2020; Wang et al. 2024). Individuals exposed to unrelieved pressure and/or shear are considered at risk, and populations such as older adults and sedated patients are particularly vulnerable to the development of pressure injuries (European Pressure Ulcer Advisory Panel et al. 2019). Among hospitalised patients, the most common types of pressure injuries are superficial, predominantly stage I and II (Li et al. 2020). More severe pressure injuries result in profound pain for patients, higher mortality risk, reduced quality of life and increased hospital costs (Burston et al. 2023; Kim et al. 2019; Nghiem et al. 2022; Song et al. 2019).

Pressure injuries experienced by patients within hospital settings are a global issue. A review of observational, cross‐sectional and longitudinal studies found an overall incidence rate of 5.4 per 10,000 patient days and a prevalence of 12.8% amongst hospitalised adults (Li et al. 2020). In Australia and New Zealand, the prevalence in acute settings was reported as 12.9%, with 7.9% being hospital‐acquired (Rodgers et al. 2021). Hospital‐acquired pressure injuries are a key indicator of the quality of care (Araujo et al. 2020; Australian Commission on Safety and Quality in Health Care 2022; Oner et al. 2021), as the majority of pressure injuries are considered avoidable. Despite systems and processes being put in place to reduce pressure injury incidence, the financial and health burden contributing to pressure injury development continues to impact both patients and the healthcare system (Australian Commission on Safety and Quality in Health Care 2022; Li et al. 2020). Given the aging global population, the incidence of pressure injuries is expected to rise, as older adults are more likely to have multimorbidity, reduced mobility and frailty, all of which contribute to an elevated risk (World Health Organization 2021).

National and international guidelines, such as those from the National Pressure Ulcer Injury Advisory Panel (NPIAP) and the European Pressure Ulcer Advisory Panel (EPUAP), recommend evidence‐based approaches to prevent and treat pressure injuries (European Pressure Ulcer Advisory Panel et al. 2019). These guidelines cover various prevention and treatment approaches recommended by clinical evidence and/or expert panels. Many strategies, such as risk assessment, skin care, repositioning, use of support surfaces and nutritional support, serve dual purposes in both prevention and treatment of pressure injuries. For example, whilst nutritional support plays a role in preventing pressure injuries, it also plays a part in promoting wound healing in existing injuries (Citty et al. 2019). Whilst the implementation of such guidelines has contributed to decreasing pressure injury rates in hospital settings over the past decade (Australian Commission on Safety and Quality in Health Care 2022; Siotos et al. 2022; VanGilder et al. 2017), the adoption and implementation of these recommended strategies in hospital settings remain challenging. Despite the widespread availability of international guidelines (European Pressure Ulcer Advisory Panel et al. 2019), there is often an evidence‐practice gap in implementing and sustaining evidence‐based clinical practice (Edsberg et al. 2022).

Implementation is the process of putting into practice or integrating evidence‐based clinical interventions within a setting (Eccles and Mittman 2006). The delivery, structure and management of healthcare services can either hinder or enable the implementation of clinical interventions (Nolte et al. 2022). Understanding how organisations effectively deliver and implement pressure injury prevention and treatment within a health service is crucial to support the translation of evidence into practice. A wide variety of organisational factors, such as nursing skill mix, readiness to change, involvement of the multidisciplinary team and inter‐professional collaboration, all have the potential to influence the implementation effort of evidence‐based clinical practices (Geerligs et al. 2021). Recognising the impact of organisational strategies is crucial, as they shape the context in which healthcare is delivered and ultimately determine the success of implementing best practices. However, in a Cochrane systematic review investigating the effectiveness of the organisation of care for the prevention and treatment of pressure injuries, only four studies were found that met inclusion criteria, indicating a significant gap in rigorous research within this field (Joyce et al. 2018). The review included hospital, residential care, aged care and rehabilitation settings and found inconclusive results regarding the impact of organisation of health care delivery on pressure injury outcomes. This might be attributed to the heterogeneity of the context and further underscores the ongoing need for comprehensive research efforts. Building on these findings, the present systematic review was conducted to update and refine some of the areas not addressed by the Joyce et al. (2018) review. The differences between this systematic review and that of Joyce et al. (2018) are outlined in Appendix S1. In the intensive care setting, Lin et al. (2020) investigated the effectiveness of multicomponent pressure injury prevention programmes (bundles) via systematic review, finding in several studies that organisational strategies focused on factors such as culture, leadership and resource allocation influenced improvement in pressure injury prevention and treatment in alignment with current evidence and guidelines. Yet, it is uncertain if these organisational strategies can be translatable or generable to other acute care hospital settings.

To date, no systematic review has been conducted recently to collate evidence on potentially effective organisational strategies for implementing pressure injury prevention and management guidelines in acute care settings. Additionally, there is a need to collate information on barriers and facilitators to implementing these strategies. This systematic review addresses these gaps by focusing exclusively on acute care settings, identifying effective organisational strategies for pressure injury prevention and management and collating information on barriers and facilitators to implementing organisational strategies. Understanding these elements might also be worthwhile to inform the development of a targeted, multi‐faceted intervention bundle to prevent or treat pressure injuries.

### 1.1 Aim

The primary aim of this review was to investigate the effects of organisational interventions on the incidence, healing and management of pressure injuries in adult patients in acute hospital settings. The scope of the review includes all organisational interventions (categorised using the Cochrane Effective Practices Of Care (EPOC) taxonomy). The secondary aim was to synthesise the evidence from the included studies on barriers and facilitators to implementing organisational strategies based on EPOC taxonomy.

The intention of this review is to provide more targeted and actionable insights into effective organisational strategies for pressure injury prevention and treatment in acute hospital settings.

## 2 Methods

### 2.1 Design

This systematic review was guided by the Cochrane Handbook for Systematic Reviews of Interventions (Higgins et al. 2023). The protocol was registered a priori with PROSPERO (CRD42023424672). Reporting follows the Preferred Reporting Items for Systematic Reviews and Meta‐analyses (PRISMA; Page et al. 2021).

The primary outcomes of interest for prevention studies were pressure injury incidence measured by rate and proportion. Incidence rate was the number of new cases during the study period, divided by the time the participant was observed. Incidence proportion was measured by the number of participants who developed a pressure injury during the study divided by the population. For treatment studies, the primary outcome was pressure injury healing, defined by the absolute or percentage change in healing rate over the time of the study. Secondary outcomes of interest were patient satisfaction and quality of life (reported using validated tools), staff satisfaction, barriers and facilitators to the implementation of interventions and adverse events. Organisational interventions were classified using the EPOC taxonomy. The EPOC group, though no longer active, developed a framework for systematic reviews of interventions aimed at improving professional practice and healthcare delivery through the evaluation of implementation strategies, service delivery, financial arrangements and governance arrangements (EPOC 2021). Through its evaluations of these strategies, EPOC aims to bridge the gap between research findings and clinical practice.

### 2.2 Search Strategy

The initial search was conducted in November 2023 and was repeated in September 2024, covering publications from 01 January 2012 to 31 December 2023. Five electronic databases were searched: Cochrane Central Register of Controlled Trials, Ovid MEDLINE, Ovid Embase, EBSCO CINAHL Complete and Web of Science Core Collection. A comprehensive literature search strategy (see Appendix S2) was developed by the reviewers in conjunction with a senior research librarian for Ovid MEDLINE and then adapted to other databases. Once studies were identified that met the criteria and were included in the review, a search of reference lists of included studies and other relevant publications such as systematic reviews and guidelines was performed. Additionally, hand searching was conducted to identify process evaluations related to the included literature. Authors of relevant studies were contacted if there was missing data from search results.

### 2.3 Inclusion Criteria

The review included published, peer‐reviewed studies with the following designs: randomised controlled trials (RCTs), non‐randomised trials, cluster RCTs, controlled before‐and‐after studies and interrupted time series studies with three data collection points, before and after the intervention on the same respondents. For studies with control groups, comparators were typically standard care. In interrupted time series studies, the analysis focuses on changes in the trend of the outcome over time, with the intervention point serving as the basis for comparison. Before‐after studies without a control and cross‐sectional studies were not included as it is difficult to attribute causation from these. Unpublished reports, conference abstracts and other forms of grey literature were excluded from this review. Only studies published in English since 2012 were included to ensure relevance to contemporary practice. This timeframe was chosen because around this time pressure injuries were increasingly recognised as key quality indicators, significant updates to pressure injury prevention guidelines and standards were being introduced, and there was a growing focus on implementation science in healthcare.

This review included studies involving hospital and patient participants, specifically targeting adults in acute hospital settings, including general medical and surgical wards, intensive care units and specialty units such as oncology or geriatrics. Studies conducted exclusively in mental health, emergency department and operating room settings were excluded. The outcome of interest was on patient‐level data for individuals at risk of developing pressure injury, identified through formal or informal risk assessments or clinical judgement, as well as those with pre‐existing pressure injury of any stage. Studies were included if interventions were aimed at improving the implementation and uptake of evidence‐based pressure injury prevention and treatment in acute hospital settings. Examples of such interventions include the use of risk assessment tools, staff training programmes and fostering interdisciplinary collaboration to influence incidence or healing rates. These strategies focus on optimising care delivery systems and adherence to best‐practice guidelines, which indirectly contribute to improved outcomes. Unlike direct clinical treatments, such as dressings or topical therapies, organisational interventions aim to enhance processes and resources that support prevention and healing rather than providing direct therapeutic effects. Inclusion criteria varied based on the type of strategy employed. Studies utilising delivery, financial or governance interventions were included if they included at least one organisation of care strategy from the EPOC taxonomy (EPOC 2021). For implementation‐focused interventions, multiple strategies had to be employed; studies using only a single implementation intervention, such as a stand‐alone educational meeting, were excluded.

### 2.4 Literature Screening and Data Extraction

Covidence systematic review software (Veritas Health Innovation 2023) was used for article screening and data extraction. Duplicates were removed, and two reviewers independently assessed study titles and abstracts to identify potentially relevant studies that met inclusion criteria. Those that did not meet inclusion criteria were excluded, with reasons recorded. Following title and abstract screening, full‐text screening was undertaken independently by two reviewers to assess for eligibility. Any disagreements were discussed and resolved by a third reviewer as necessary. Data were extracted from the included studies using a standardised form that captured essential information, including study characteristics (author, title, year, country of origin, study design, setting, method and level of allocation, recruitment, intervention period, follow up, funding source, ethical approval, etc.), intervention characteristics (EPOC taxonomy components), frameworks guiding intervention or implementation, reported barriers and facilitators to implementation and outcomes. Two reviewers independently conducted data extraction. Disagreements about the data extracted were resolved through discussion between the reviewers, and where needed, a third reviewer was consulted to resolve discrepancies.

### 2.5 Assessment of Risk of Bias

Two reviewers independently assessed risk of bias, with disagreements adjudicated through a third reviewer. The risk of bias tool for cluster‐randomised trials (RoB 2 CRT) was applied to the cluster trials, assessing six domains, including identification bias—a major challenge in cluster trials (Higgins et al. 2022)—which is not included with the standard RoB 2 tool for RCTs (Higgins et al. 2022; Sterne et al. 2019). For non‐randomised studies, bias was assessed using the Risk Of Bias In Non‐randomised Studies of Interventions (ROBINS‐I) tool (Sterne et al. 2016), which is comprised of seven domains.

### 2.6 Data Synthesis

A narrative synthesis was employed. Given the heterogeneity of study designs and outcomes, a meta‐analysis was not possible. The synthesis was structured around three main themes: types of interventions used, theoretical frameworks used and barriers and facilitators to implementation reported. Within each theme, findings were differentiated between randomised and non‐randomised studies to facilitate comparison across study designs.

### 2.7 Certainty of the Body of Evidence

The certainty of evidence was assessed using the Grading of Recommendations, Assessment, Development and Evaluation (GRADE) approach (Balshem et al. 2011). This method can be applied even when meta‐analysis is not feasible due to heterogeneity in outcome measurements or reporting across studies (Murad et al. 2017). The GRADE approach is used to evaluate the certainty of evidence based on methodological limitations of the studies, indirectness, imprecision, inconsistency and publication bias. An overall certainty rating of high, moderate, low or very low was given. The certainty can be downgraded or upgraded based on the assessment of the five domains.

## 3 Results

### 3.1 Study Selection

**FIGURE 1:**
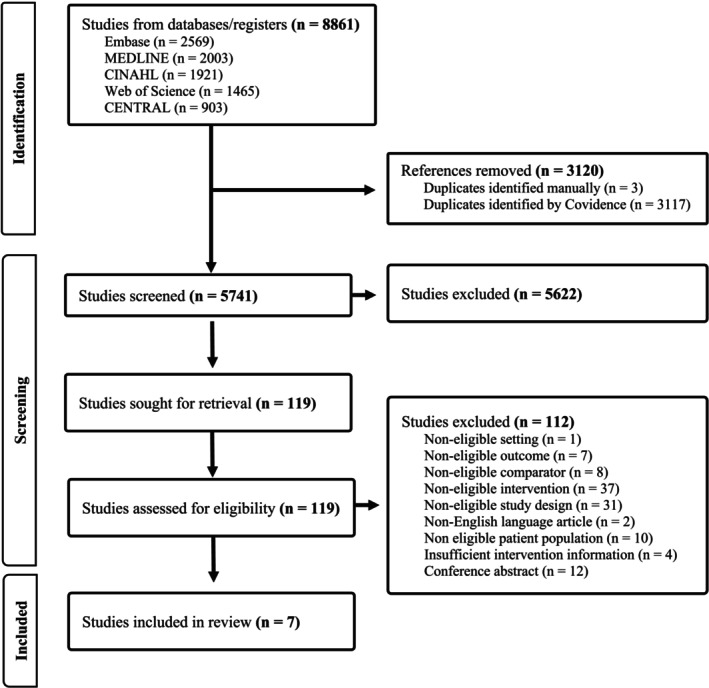
PRISMA flow diagram.

The initial search strategy was first conducted on 03 November 2023, covering the period from 01 January 2012 to 03 November 2023. To ensure comprehensive coverage, including articles that might have been catalogued with a delay, the authors conducted a follow‐up search in September 2024, extending the coverage period to December 31, 2023. This two‐step approach was designed to capture all relevant literature within the timeframe of 2012–2023. From these two searches, a total of 8861 articles were retrieved. After removing 3120 duplicates, 5741 abstracts remained. Following the screening of titles and abstracts, 119 full texts were reviewed independently by two reviewers. Of these, seven studies met eligibility criteria and were included in the final review (Baghaei and Azar 2016; Chaboyer et al. 2016; Jafary et al. 2018; Mudge et al. 2022; Padula et al. 2015; Pol‐Castaneda et al. 2022; Tayyib et al. 2015). The PRISMA flow diagram is shown in Figure 1.

### 3.2 Study Characteristics

The characteristics of the included studies are summarised in Table 1. The seven studies were conducted in five countries. Four studies employed a cluster randomised controlled design (Chaboyer et al. 2016; Jafary et al. 2018; Mudge et al. 2022; Tayyib et al. 2015), of which Tayyib et al. (2015) used a stepped‐wedge approach, which involved random sequential crossover of clusters from control to intervention. Three were non‐randomised studies, of which one was a non‐randomised study of an intervention with historical control (Baghaei and Azar 2016). Although the authors described using randomisation, insufficient information was provided regarding randomisation and allocation methods to evidence its inclusion as a truly randomised study. Padula et al. (2015) employed a quasi‐experimental interrupted time series design and Pol‐Castaneda et al. (2022) conducted a parallel group quasi‐experimental controlled study.

**TABLE 1:** Study characteristics.

Authors, year, country	Design	Setting and participants	Sample size	Data collection dates	Follow up period	Funding
Baghaei and Azar (2016), Iran	Non‐randomised intervention with historical controls	Two hospitals; ICU; high‐risk patients	Intervention *n* = 250, control *n* = 250	NR	3 months	NR
Chaboyer et al. (2016), Australia	Cluster‐randomised controlled trial	Eight hospitals; surgical, medical, cancer; at‐risk adult patients	Intervention *n* = 799, control *n* = 799	June 2014–May 2015	11 months	National Health and Medical Research Council
Jafary et al. (2018), Iran	Stepped‐wedge cluster‐randomised controlled trial	One hospital; 16 units: internal, surgical, ICU; high‐risk patients	Intervention *n* = 1657, control *n* = 1855	June 2015–November 2016	10 months (45 weeks)	Endocrinology and Metabolism Clinical Sciences Institute
Mudge et al. (2022), Australia	Cluster‐randomised controlled trial	Four hospitals; eight acute medical and surgical wards; patients ≥ 65 years	Intervention *n* = 265, control *n* = 274	October 2016–April 2017	6 months	Queensland Accelerate Partnership Grant
Padula et al. (2015), USA	Quasi‐experimental interrupted time series	55 medical centres; all patients	Intervention *n* = 1,590,022	September 2007–February 2012	60 months	NR
Pol‐Castaneda et al. (2022), Spain	Parallel group quasi‐experimental controlled study	Three hospitals; medical and surgical units; all adult patients	Intervention *n* = 1797, control *n* = 1945	April 2018–September 2019	12 months	Nursing College of the Balearic Islands
Tayyib et al. (2015), Kingdom of Saudi Arabia	Cluster‐randomised controlled trial	Two hospitals; ICU; adult patients	Intervention *n* = 70, control *n* = 70	October 2013–February 2014	28 days	NR

Abbreviation: NR, not reported.

Four studies reported using a theoretical framework to either guide development and/or implementation of the interventions. Of the randomised studies, Chaboyer et al. (2016) reported use of the Medical Research Council framework for the development of complex interventions (Craig et al. 2008) to guide development of the study. Two studies (Mudge et al. 2022; Pol‐Castaneda et al. 2022) used the integrated‐Promoting Action on Research Implementation in Health Services (i‐PARIHS) (Harvey and Kitson 2016) to guide and identify elements to design and implement the study, with the latter study also using Ajzen's (1991) Theory of Planned Behaviour to understand nurse behaviour change processes. A conceptual framework for implementation science (Gonzales et al. 2012) was used by Padula et al. (2015) in their interrupted time series study to explain events that occurred between policy, evidence‐based practice and quality improvements for hospital‐acquired pressure injury prevention.

The total sample size of the seven studies was 1,600,339 (range 140–1,590,022). The overall sample sizes of the four cluster RCTs were 6075; with 49.3% (*n* = 2998; range 70–1855) in the control groups and 45.9% (*n* = 2791; range 79–1855) in the intervention groups. One study (Jafary et al. 2018) employed a stepped‐wedge design, which included 286 participants in the total sample size, though it was considered to be in a training period. In six studies, participants were recruited from multiple hospitals; however, the single‐site study recruited from 16 units within that one hospital (Jafary et al. 2018). Three studies recruited all hospital patients (Padula et al. 2015; Pol‐Castaneda et al. 2022; Tayyib et al. 2015) and one was limited to older patients (≥ 65 years of age; Mudge et al. 2022). Two studies included only patients at high risk of developing pressure injury (Baghaei and Azar 2016; Jafary et al. 2018) and one recruited all at‐risk patients identified by limited mobility (Chaboyer et al. 2016). All studies required participant informed consent and reported receiving ethics approval. Four studies reported receiving funding, with no conflicts of interest reported (Chaboyer et al. 2016; Jafary et al. 2018; Mudge et al. 2022; Pol‐Castaneda et al. 2022).

### 3.3 Risk of Bias

Of the four cluster‐randomised studies, the overall risk of bias was low in one (Chaboyer et al. 2016), held some concerns in two (Mudge et al. 2022; Tayyib et al. 2015) and was high in the other (Jafary et al. 2018; Table 2a). Cluster RCTs with pre‐registered protocols, such as Chaboyer et al. (2016) and Mudge et al. (2022) generally had a lower risk of bias.

**TABLE 2a:** Risk of bias for cluster‐randomised trials (RoB 2 CRT).

Risk of bias domains	Authors			
	Chaboyer et al. (2016)	Jafary et al. (2018)	Mudge et al. (2022)	Tayyib et al. (2015)
1a: Bias arising from the randomisation process	Low	Low	Low	Low
1b. Bias arising from the identification or recruitment of participants into clusters	Low	Low	Low	Low
2: Bias due to deviations from the intended interventions	Low	Some concerns	Some concerns	Some concerns
3: Bias due to missing outcome data	Low	Low	Low	Low
4: Bias in measurement of the outcome	Low	High	Low	Some concerns
5: Bias in selection of the reported results	Low	High	Some concerns	Some concerns
Overall risk of bias	Low	High	Some concerns	Some concerns

The use of pre‐registered protocols impacted the risk of bias judgements across several domains, including deviations from intended interventions, measurement of outcomes and selection of reported results. Cluster RCTs demonstrated a low risk of bias in randomisation, employing methods such as random number generation (Chaboyer et al. 2016), coin toss (Mudge et al. 2022) and computer‐generated randomisations (Tayyib et al. 2015). However, blinding of interventions for cluster‐randomised trials proved difficult due to the nature of the interventions. Chaboyer et al. (2016) implemented strategies to reduce bias, including blinding hospital staff to comparator interventions and group allocation and masking outcome assessors to trial hypothesis, design and allocation. Chaboyer et al. (2016) blinded both the statistician and patients to group allocation, although patients were aware they were in a study examining pressure injury prevention strategies. In contrast, Jafary et al. (2018) made no attempts to blind interventions, and the absence of a protocol made it difficult to assess bias in selective reporting. Whilst two nurses collected data without carrying out extra interventions, this did not sufficiently mitigate the overall high risk of bias, particularly in the measurement of outcomes and selection of reported results (Jafary et al. 2018).

The risk of bias in the non‐randomised studies was serious in two (Padula et al. 2015; Pol‐Castaneda et al. 2022) and critical in one (Baghaei and Azar 2016; Table 2b). Methodological limitations contributed to a critical risk of bias in Baghaei and Azar (2016), including the absence of an analysis plan, unreported missing data and disproportionate participant exclusions in the intervention group. Similarly, Pol‐Castaneda et al. (2022) was judged to have serious bias concerns, as missing data and pre‐intervention data were not reported despite being outlined in the pre‐registered protocol, impacting the selection of reported results.

**TABLE 2b:** Risk of bias in non‐randomised studies of interventions (ROBINS‐I).

Risk of bias domains	Authors		
	Baghaei and Azar (2016)	Padula et al. (2015)	Pol‐Castaneda et al. (2022)
1: Bias due to confounding	Serious	Serious	Moderate
2: Bias in selection of participants into the study	Serious	Moderate	Low
3: Bias in classification of interventions	Moderate	Serious	Low
4: Bias due to deviations from intended interventions	Moderate	Serious	Low
5: Bias due to missing data	Critical	No information	No information
6: Bias in measurement of outcome	Serious	Serious	Serious
7: Bias in selection of the reported result	Moderate	Moderate	Serious
Overall risk of bias	Critical	Serious	Serious

Overall, the use of pre‐registered protocols and robust randomisation methods reduced bias in cluster RCTs, whilst non‐randomised studies faced methodological challenges that increased bias from missing data and selective reporting.

### 3.4 Types of Intervention

The intervention components using the EPOC classification, theoretical frameworks supporting the development or implementation of the interventions, barriers and enablers and outcomes of the intervention are summarised in Table 3. Table 4 provides a concise overview of the intervention components used across studies. Between three and eight implementation strategies were employed in each study, and between one and two service delivery arrangements were used in four studies (Chaboyer et al. 2016; Mudge et al. 2022; Padula et al. 2015; Pol‐Castaneda et al. 2022). Notably, no financial or governance arrangements were used in the reviewed studies.

**TABLE 3:** Intervention components and outcomes.

Authors	EPOC intervention components		Development/implementation framework	Barrier and facilitators	Pressure injury incidence
	Implementation strategies	Delivery arrangements			
Baghaei and Azar (2016)	*Education materials*: Educational booklet including general information of pressure injury was prepared by the researcher and is distributed amongst the two hospital's nurses. In the provided booklet pressure injury is comprehensively reviewed and required information on the prevention of pressure injury explained for nurses. Showing films. Flowcharts in the form of banners. *Educational meetings*: Guideline training was performed by holding workshops. *Educational outreach*: Practical instruction at the patients' bedsides. The correct way of transferring, diagnosing various stages of pressure injury and monitoring the skin was explained to the nurses at the patients' bedsides	NR	NR	NR	Intervention 32.8% (82/250); control 53.6% (134/250); *p* < 0.001
Chaboyer et al. (2016)	*Educational materials*: information brochures, 5‐min DVD and patient posters; PowerPoint presentations and resources provided to nurses. *Educational meetings*: Four to eight formal sessions conducted prior to and during data collection. *Educational outreach*: Face‐to‐face education was provided to patients at their bedside. *Patient‐mediated interventions*: Patient received intervention from intervention research assistant; patient watched DVD, reviewed brochure and chose where to display poster	*Self‐management*: Resources used to educate patient and encourage participation in pressure injury prevention	Medical Research Council Framework for the development of complex interventions	*Barriers*: Patients—no top up dose of intervention, low perceived importance of pressure injury prevention, trouble retaining information, lack of interaction in learning. Nurses—no top up dose of intervention *Enablers*: Patients—human interaction important in facilitating engagement with bundle delivery, outcome assessors acted as reminders for patients, understanding pressure injury prevention enhances participation, knowledge and awareness of pressure injury prevention motivated patients to participant, partnering with nurses improves maintenance issues. Nurses: leadership, influence and intervention being simple to deliver enabled implementation and sustainment into practice; Patients and nurses responded well when they found it to be low complexity, aligning with current knowledge and seeing the benefits to the bundle	Intervention: 6.1% (49/799); control: 10.5% (84/799); cluster adjusted *p* = 0.644
Jafary et al. (2018)	*Educational materials*: Pamphlets and posters. *Educational meetings*: Training session for clinical staff. *Educational outreach*: Daily visits by pressure injury expert as routine hospital care for high‐risk patients. *Reminders*: Bedside high‐risk cards targeted at clinicians	NR	NR	NR	Cumulative incidence: intervention 9.5% (158/1657); control: 10.9% (203/1855)
Mudge et al. (2022)	*Audit and feedback*: Structured audits of care processes to inform opportunities for improvement. *Communities of practice &* *Continuous quality improvement*: Site facilitators engaged staff from the intervention ward to form a multidisciplinary working group that reviewed local interview and audit findings to prioritise areas for improvement aligned with the key principles and programme goals. *Local opinion leaders*: Multidisciplinary working group key stakeholders and champions (e.g., nurse unit managers, nurse educator, physiotherapist, dietitian and occupational therapist) met for 1 h per month. The group collaborated with the facilitator and other staff as needed to implement iterative improvements. *Tailored interventions*: Interventions to change practice were based on identifying local barriers and enablers through structured patient interviews and structured audits of care processes	*Role expansion or task shifting*: A nurse or allied health professional from within each hospital was employed for 2 days per week as a site facilitator, trained and mentored by two experienced facilitators	i‐PARIHS implementation framework	*Barriers*: High‐perceived nursing workload; task‐centred nursing culture; limited team communication; limited nurse unit manager, allied health or medical leadership; scarce chairs; previous imposed change; crowded bays; limited group spaces *Enablers*: Strong nurse unit manager, allied health and medical leadership; previous clinician‐led change; person centred culture; strong communication; previous successful organisational change; culture supporting change; regular use of group spaces; regular use of patient group spaces	Intervention 6.8% (18/265); control 6.6% (18/274); OR 1.56 (95% CI 0.73–3.31)
Padula et al. (2015)	*Monitoring*: Data tracking, analysis and dissemination of pressure injury rates within units and hospitals (e.g., data wall displays); participation in a benchmarking project to assess baseline rates of pressure injury outcomes. *Communities of practice*: Prevention Awareness: A cooperative ongoing effort amongst a multidisciplinary committee (e.g., nurses and physicians) in prevention awareness. *Continuous quality improvement*: Wound/quality improvement team; established wound care clinician approach/teamwork with quality improvement people (e.g., review of epidemiology data; quality reporting; shared leadership). *Educational meetings*: Prevention Education: Continuing education about prevention; Staff Training: Training and orientation for new or unfamiliar staff; All‐staff meetings: Frequent all‐staff (town hall) meetings to discuss prevention guidelines. *Clinical practice guidelines*: Prevention protocol: incorporated pressure injury prevention protocol into institutional policies and procedures. *Reminders*: Visual reinforcement tools, e.g., checklists, posters or bundled interventions for follow‐through with prevention protocols	*Teams*: Established multidisciplinary wound care team for pressure injury prevention *Health information systems*: A standardised risk assessment tool was built into the Electronic Health Record system; electronic trigger/alarm system related to pressure injury risk	A conceptual framework for implementation science	NR	Timepoint 1: 1.4% (1085/76,929); Timepoint 2: 0.8% (2575/321,969); Timepoint 3: 0.9% (590/310,690); Timepoint 4: 0.2% (607/319,318); Timepoint 5: 0.1% (336/373,190); Timepoint 6: 0.8% (150/187,926)
Pol‐Castaneda et al. (2022)	*Audit and feedback*: Independent audits were conducted to assess issues related to the prevention and care of pressure injury. *Monitoring*: All APHNs were monitored and advised by the research team via individual contact as necessary and at monthly meetings between the research team, the APHNs, the support nurses and the supervisors. *Communities of practice*: The APHN group, support nurses and supervisors met regularly (initially weekly, then monthly) to assess progress, share materials and experiences and ensure accurate data collection, in collaboration with the research team. *Educational meetings*: After selection of hospital wards, project information meetings were held for the nursing teams, who were invited to participate. *Clinical practice guidelines*: The APHN designed specific actions to put into effect two clinical practice guidelines. *Local opinion leaders*: Two nurses were selected from each unit (one APHN and one support nurse) who received specified training. *Tailored interventions*: The APHNs designed specific actions to put into effect two clinical practice guidelines, adapted as appropriate to the specific context and its characteristics	*Role expansion or task shifting*: Incorporation of an APHN within each unit involved, to participate in activities appropriate to the context in question, with specific interest in providing support to health teams, motivating attitudinal change regarding skills, abilities and knowledge and seeking to ensure the implementation of CPGs recommendations and the avoidance of low‐value practices. A second nurse from the team (the ‘support RN’) was also selected to stand in for the primary APHN if necessary	i‐PARIHS implementation framework; Theory of Planned Behaviour	NR	Baseline: intervention 7.9% (2/151); control 8.8% (14/160). Post‐intervention (month 12): intervention 5.5% (7/126); control 6.6% (10/151) Cumulative incidence: intervention 5.7% (103/1797); control 8.7% (169/1945); *p* < 0.001
Tayyib et al. (2015)	*Audit and feedback*: RN compliance to the intervention was audited monthly and feedback was provided. *Continuous quality improvement*: Consultation and clarification with the researcher, which continued throughout the study. *Educational materials*: Brochures explaining the elements of the pressure injury prevention bundle. *Educational meetings*: In‐service meetings; PowerPoint presentation with a handout used during in‐service. *Educational outreach*: One‐to‐one bedside education provided by the researcher	NR	NR	NR	Intervention 17.1% (12/70); control 52.9% (37/70); *p* < 0.001

Abbreviations: APHN, advanced practice hospitalisation nurse; CPG, clinical practice guidelines; CRT, cluster‐randomised trial; NR, not reported; NRT, non‐randomised trial; RN, registered nurse.

**TABLE 4:**
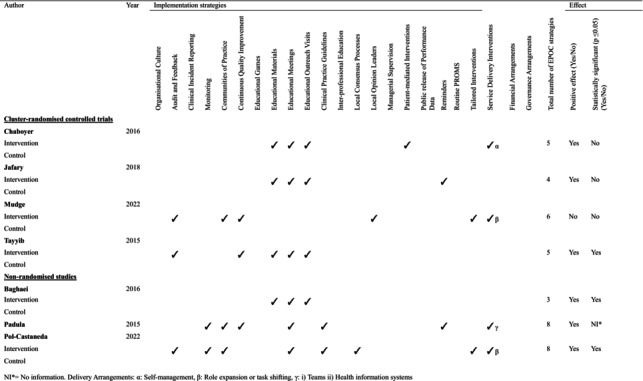
Summary of EPOC organisational interventions.

*Note:* Delivery Arrangements: α: self‐management; β: role expansion or task shifting; γ: (i) teams and (ii) health information systems.

Abbreviation: NI, no information.

The most commonly reported implementation strategies were educational (*n* = 6), including the use of educational materials (Baghaei and Azar 2016; Chaboyer et al. 2016; Jafary et al. 2018; Tayyib et al. 2015), meetings (Baghaei and Azar 2016; Chaboyer et al. 2016; Jafary et al. 2018; Padula et al. 2015; Pol‐Castaneda et al. 2022; Tayyib et al. 2015) and outreach visits (Baghaei and Azar 2016; Chaboyer et al. 2016; Jafary et al. 2018; Tayyib et al. 2015), audit and feedback (*n* = 3) (Mudge et al. 2022; Pol‐Castaneda et al. 2022; Tayyib et al. 2015), communities of practice (*n* = 3) (Mudge et al. 2022; Padula et al. 2015; Pol‐Castaneda et al. 2022) and continuous quality improvement (*n* = 3) (Mudge et al. 2022; Padula et al. 2015; Tayyib et al. 2015). These strategies were directed at patients and clinicians in acute hospital settings, primarily nurses. When interventions targeted patients, education strategies were primarily used to promote patients' participation in pressure injury prevention practices and to ensure their adoption. For nurses providing direct patient care, interventions included face‐to‐face education, takeaway educational materials and practical demonstrations of how to apply knowledge. Strategies aimed at enhancing clinical practices often focused on systematic approaches to identify areas for improvement and engage key stakeholders. Across the included studies, common approaches included data collection and analysis to pinpoint specific care practices that required enhancement, as well as the identification of influential individuals and groups within the hospital setting.

### 3.5 Study Outcomes

All included studies focused solely on the prevention of pressure injuries with incidence as the main outcome. None were focused on the treatment of pressure injury or healing rates. With the exception of the study by Mudge et al. (2022), all studies reported a reduction in pressure injury incidence. The differences were statistically significant in three studies (Baghaei and Azar 2016; Pol‐Castañeda et al. 2020; Tayyib et al. 2015). In the largest study (Padula et al. 2015), although there was a gradual fall in pressure incidence across six time points, the initial rate was very low (time 1: 1.4%). None of the included studies reported on secondary outcomes such as patient satisfaction, quality of life (measured using validated tools) or staff satisfaction. Additionally, no study explicitly reported on adverse events related to the interventions.

### 3.6 Barriers and Facilitators

After further hand‐searching of process evaluation papers relevant to the seven included studies, barriers and facilitators were found reported in the subsequent process evaluation papers (Mudge et al. 2023; Roberts et al. 2017) of the two included randomised studies (Chaboyer et al. 2016; Mudge et al. 2022). The identified barriers and facilitators were found to be associated with both the implementation of organisational strategies and clinical interventions for pressure injury prevention, as reported in the included studies. Common barriers and facilitators (see Table 3) across the two evaluations included highlighting the importance of strong nursing leadership as an enabler and lack of leadership as a barrier to successful practice change. Examples of barriers reported by patients in one study (Chaboyer et al. 2016) included lack of top‐up dose of intervention—referring to insufficient follow‐up or reinforcement of the initial intervention—as well as lack of interaction in learning and low perceived importance of pressure injury prevention (Roberts et al. 2017). Nurses provided insights on how leadership, influence and keeping the intervention simple enabled implementation and sustaining in practice (Roberts et al. 2017). Similarly, strong nurse unit manager, allied health professional and medical leadership were reported as facilitators, and limited leadership was reported as a barrier to the implementation of a multicomponent ward‐based improvement intervention (Mudge et al. 2022). Barriers to this study included a high‐perceived nursing workload, a task‐centred culture, limited team communication, imposed change and environmental factors (Mudge et al. 2023). Environmental factors in this context referred to physical aspects of the hospital environment that could impact the implementation of pressure injury prevention strategies, such as ward layout, availability of equipment and other elements of the physical workspace that might facilitate or hinder the intervention.

### 3.7 Certainty of Evidence on Pressure Injury Incidence

#### 3.7.1 Methodological Limitations

Serious concerns were noted due to unclear randomisation procedures in some RCTs and a high risk of bias in non‐randomised studies. Whilst large sample sizes in studies like Pol‐Castaneda et al. (2022) and Padula et al. (2015) add weight to their findings, these studies also had significant risks of bias, particularly related to deviations from intended interventions. This bias is likely to overestimate the true effect of the interventions on outcomes.

#### 3.7.2 Indirectness

Some concerns were noted regarding indirectness. Whilst the populations, comparators and outcomes were directly relevant to our review question, the distinction between the effects related to clinical interventions and organisational strategies was often unclear due to not well‐defined routine care in the control arm. The inclusion of the effects of implementing clinical interventions may likely overestimate the true effect of organisational strategies.

#### 3.7.3 Imprecision

Serious concerns were noted. Individual study samples ranged from 140 (Tayyib et al. 2015) to over 1.5 million (Padula et al. 2015), with most studies exceeding 400 participants. However, confidence intervals were inconsistently reported, and where they were, such as Mudge et al. (2022) (OR 1.56, 95% CI 0.73–3.31), the intervals were wide.

#### 3.7.4 Inconsistency

Serious concerns were noted due to the heterogeneity in effectiveness. Three studies reported statistically significant reductions in pressure injury incidence (Baghaei and Azar 2016; Pol‐Castañeda et al. 2020; Tayyib et al. 2015), whilst others showed no significant effect (Chaboyer et al. 2016; Jafary et al. 2018) or even a slight increase in incidence, though not statistically significant (Mudge et al. 2022). Padula et al. (2015) reported varying results across different timepoints.

#### 3.7.5 Publication Bias

The comprehensive search strategy and the mix of positive and negative findings in the included studies suggest that publication bias was not strongly suspected.

#### 3.7.6 Overall Assessment

The overall certainty of evidence is rated as low, given the presence of serious concerns in three domains (methodological limitations, imprecision and inconsistency) and some concerns in the indirectness domain.

## 4 Discussion

### 4.1 Principal Findings

The findings of this systematic review were that multiple organisational strategies targeting clinicians and patients may reduce the incidence of pressure injuries in acute hospital settings. Yet, the specific combination of organisational strategies within the multicomponent approaches remains uncertain. The seven studies included described interventions aimed at preventing pressure injuries, all of which utilised various strategies to change clinician and patient behaviour and practice within acute hospital environments. Overall, whilst all but one of the studies reported a positive effect on pressure injury reduction, only three were statistically significant. The variability in the design, implementation strategies and outcomes used across studies makes it challenging to identify an optimal combination of interventions for reducing pressure injury incidence.

In our review there was variability in the effectiveness of included interventions, with only three studies reporting statistically significant results (Baghaei and Azar 2016; Pol‐Castaneda et al. 2022; Tayyib et al. 2015) with all three found to be at risk of bias to some degree. Furthermore, the setting for two of these studies was intensive care (Baghaei and Azar 2016; Tayyib et al. 2015), where patients are generally at greater risk of pressure injury. In an earlier systematic review of organisation strategies to prevent pressure injury only four studies met inclusion criteria (Joyce et al. 2018). Their review encompassed a broad range of organisational strategies but reported very low certainty of evidence for all interventions. It was unclear in three of the studies (Bloemen‐Vrencken et al. 2007; Caplan et al. 1999; Stern et al. 2014) whether the intervention had any effect on reducing pressure injuries, and it was unclear whether the intervention had any effect on pressure injury healing in the other (Vu et al. 2007). As well, none of the interventions were implemented within an acute hospital setting.

The heterogeneity of interventions across studies reveals a significant challenge in pressure injury prevention research. Whilst all studies included in this review employed multiple interventions, the lack of commonality across studies limits the ability to draw conclusions about the effectiveness of the various organisational strategies or their combinations. This variability suggests that pressure injury prevention is a complex, multifaceted issue that may not have a one‐size‐fits‐all solution. The trend towards multicomponent and person‐centred care bundles, as highlighted by Chaboyer et al. (2024), reflects a shift towards comprehensive, individualised strategies addressing various risk factors associated with pressure injuries. Within these multicomponent interventions, key clinical interventions typically include regular skin assessments, repositioning, use of pressure‐relieving devices and nutritional support.

The studies in this review reflected this variety of approaches, with different combinations of these key clinical interventions. Skin assessment was explicitly mentioned in studies by Pol‐Castaneda et al. (2022) and Baghaei and Azar (2016). Repositioning was a key clinical intervention in several studies (Baghaei and Azar 2016; Jafary et al. 2018; Padula et al. 2015; Pol‐Castaneda et al. 2022). Pressure‐relieving devices were utilised in studies by Jafary et al. (2018) and Baghaei and Azar (2016). Nutritional support was addressed by Pol‐Castaneda et al. (2022), Mudge et al. (2022), Padula et al. (2015) and Baghaei and Azar (2016). Despite the critical role these clinical interventions play in pressure injury prevention bundles, this review reveals significant gaps in understanding how organisational strategies can effectively support the implementation of these clinical interventions.

One large cohort study of 1801 acute care facilities in the United States indicated that compliance with skin assessments and pressure redistribution is generally high, but there was room for improvement in the implementation of some of the most fundamental prevention strategies, including repositioning, heel elevation, nutritional support and moisture management (Edsberg et al. 2022). Whilst the implementation of pressure‐relieving surfaces and timely repositioning has been shown to significantly reduce hospital‐acquired pressure injuries (European Pressure Ulcer Advisory Panel et al. 2019), challenges like inadequate staff education and limited capacity to adhere to pressure injury guidelines can hinder the effectiveness of these interventions (Wan et al. 2023). Crucially, the limited standardised reporting on the implementation strategies complicates the identification of effective components, underscoring the importance of process evaluation in clinical trials. These trials and evaluations should measure implementation outcomes such as acceptability, adoption, appropriateness, cost, feasibility, fidelity, penetration and sustainability to gauge how effectively evidence‐based practices are implemented into clinical settings (Mettert et al. 2020). For example, Lavallee et al. (2019) conducted a feasibility study to reduce pressure injuries in nursing homes and found that intervention fidelity was only 16%, highlighting the difficulty in measuring the true intervention effects. Low fidelity can obscure potential benefits, leading to underestimation of effectiveness or misidentification of key components contributing to pressure injury reduction. By incorporating process evaluations, future research can help to better understand how multicomponent organisational strategies effectively reduce pressure injury incidence, ensure interventions are delivered as intended and facilitate context‐specific adaptations. This approach will enhance the applicability of pressure injury prevention interventions across diverse clinical contexts.

### 4.2 Clinical and Research Implications

This systematic review demonstrated that organisational strategies involving multicomponent implementation strategies and service delivery arrangements may effectively reduce pressure injury incidence in acute hospital settings. However, it also revealed variability in the implementation strategies and outcomes across studies, making it challenging to identify an optimal combination of strategies for reducing pressure injury incidence. From a clinical perspective, the review highlights the diversity of implementation strategies that can be employed to translate evidence into practice for pressure injury prevention. These strategies ranged from educational approaches and audit and feedback to reminders and communities of practice. This variety suggests that healthcare providers have multiple options for implementing evidence‐based practices, and the choice of strategy may depend on the specific context and resources of their healthcare setting.

Our findings indicate that organisations in the reviewed studies invested in multifaceted pressure injury prevention programmes. These programmes often included various strategies targeting care delivery and staff engagement, reflecting the diverse contexts in which they were implemented. This variability suggests that understanding local needs and conditions is crucial for effectively translating evidence into practice, as different settings may require unique combinations of interventions to achieve optimal outcomes. The review highlighted the use of multiple implementation strategies and service delivery arrangements in pressure injury prevention efforts, though their comparative effectiveness to single interventions was not directly assessed. Several studies used theoretical frameworks to guide intervention implementation. Nilsen et al. (2020) argue that the use of theoretical frameworks can enhance the understanding of the implementation processes and outcomes, highlighting the need for consistent application of such frameworks in future pressure injury prevention research.

The risk of bias results from this review indicate a need for more robust studies in the future to compare the effectiveness of different combinations of implementation strategies, as well as evaluating single versus multiple intervention approaches. There is a need to explore the impact of organisational factors on intervention success and to investigate methods for engaging multiple stakeholders in pressure injury prevention and treatment efforts. Addressing these challenges requires methodological advancements. Traditional parallel controlled designs often face difficulties in health services research, particularly due to site reluctance to serve as non‐intervention controls. This reluctance stems in part from ethical concerns about withholding potential beneficial interventions from participants in control groups, especially when existing practices are known to be suboptimal (Prost et al. 2015). These ethical challenges may discourage the use of traditional parallel controlled designs in health services research, contributing to the limited number of trials meeting inclusion criteria for this review. Modern approaches, such as stepped‐wedge designs, offer a practical solution by allowing all sites to receive the intervention whilst acting as their own controls during phased implementation (Hemming et al. 2015). This design addresses concerns of withholding interventions from control sites, promotes multiple sites participation in research studies and allows researchers to understand how interventions work over time and in varied settings. Furthermore, incorporating pre‐registered protocols with detailed analysis plans and blinded outcome assessors can enhance the rigour of future studies. By adopting these methodological improvements, researchers can reduce bias, improve transparency and ensure reproducibility in research projects. These enhancements are crucial for building robust evidence that supports decision making.

### 4.3 Strengths and Limitations

The inclusion of both randomised and non‐randomised controlled studies in this systematic review was critical to capture the limited evidence on organisational strategies for pressure injury prevention. The inclusion of only controlled studies enhances the reliability of the findings by providing a basis for comparison. Quasi‐experimental designs reflect real‐world applicability, often the only feasible evaluation method due to the high cost of randomised trials (Cham et al. 2024).

The targeted focus on acute hospital settings allows for a deeper understanding of effective strategies in these settings but limits the generalisability of the findings to within hospital settings. The use of the EPOC classification system provided a standardised approach to categorising intervention components, enhancing comparability across studies. Additionally, the review's focus on theoretical frameworks and implementation strategies aligns with current evidence in implementation science, offering valuable insights into the mechanisms of successful healthcare intervention implementation and sustainability.

Limitations of this review included the heterogeneity of study designs and interventions made direct comparisons challenging and precluded meta‐analysis. The inclusion of studies with varying levels of bias risk may have impacted the overall quality of evidence; however, the use of specific tools for assessing randomised and non‐randomised studies provides an accurate reflection of these bias levels. Furthermore, the lack of reporting on barriers and facilitators in the non‐randomised studies limited the understanding of contextual factors in these settings. Lastly, the review also yielded only studies focusing on prevention and not treatment, which may narrow the scope of the findings.

## 5 Conclusion

The findings of this systematic review suggest that organisational strategies incorporating multiple implementation strategies and service delivery arrangements may potentially reduce pressure injury incidence in acute hospital settings. Whilst they are inconclusive overall, our findings suggest that interventions incorporating multiple implementation strategies and service delivery arrangements may potentially reduce pressure injury incidence. However, the variability in study designs, intervention components and outcomes across the seven included studies makes it challenging to identify optimal intervention combinations. Whilst this review offers valuable insights, it highlights the need for more rigorous research to identify effective organisational interventions and improve the reporting of implementation outcomes in pressure injury prevention efforts.

## Author Contributions

**Jake McMahon:** writing – original draft, writing – reviewing and editing, visualisation, methodology, project administration, data curation, formal analysis, conceptualisation. **Elizabeth McInnes:** writing – reviewing and editing, supervision, methodology, data curation, formal analysis, conceptualisation. **Ching Shan Wan:** writing – reviewing and editing, methodology, data curation, formal analysis. **Nicola Straiton:** writing – reviewing and editing, data curation, formal analysis. **Louisa Lam:** writing – reviewing and editing, supervision, data curation. **Jane Rodgers:** writing – reviewing and editing. **Jessica Dickson:** writing – reviewing and editing, methodology, data curation. **Paul Fulbrook:** writing – reviewing and editing, supervision, methodology, data curation, formal analysis. All authors have made substantial contributions to the work, revised it critically, approved the final version and agreed to be accountable for its accuracy and integrity.

## Ethics Statement

The authors have nothing to report.

## Conflicts of Interest

Elizabeth McInnes was an author of one of the included studies in the systematic review. She was not involved in data extraction, risk of bias assessment or analysis of the included study. All remaining authors declare no conflicts of interest.

## Supporting information


Appendix S1.

Appendix S2.

Data S1.

## Data Availability

The authors have nothing to report.
