# Supplementary material for: Effectiveness of Organisational Strategies for Pressure Injury Prevention and Treatment in Acute Hospital Settings: A Systematic Review
Source: J Adv Nurs. 2025 Jun 5;82(3):2004–21. doi: 10.1111/jan.17090 (PMC12907601; doi:10.1111/jan.17090)
Supplement: Supplementary file 1 — Appendix S1. [file JAN-82-2004-s003.docx]

Appendix A

***Differences between Joyce et al review and current review***

| **Study eligibility criteria** | **Original (**[**Joyce et al., 2018**](#_ENREF_23)**) review** | **Current review** |
| --- | --- | --- |
| Aim | To assess the effects of different provider‐orientated interventions targeted at the organisation of health services, on the prevention and treatment of pressure ulcers. | To assess the effectiveness of healthcare organisational delivery strategies to prevent and treat pressure injuries in acute care settings. |
| Dates searched | No date – April 2018 | January 2012 – December 2023 |
| Types of studies | RCTs, cluster-RCTs, non-RCTs, controlled before-and-after studies, interrupted time series studies | |
| Types of participants | Any age in any care setting (hospitals, nursing homes, residential care, rehabilitation centres) | Adults over 18 years in acute care settings |
| Types of interventions | Provider-orientated interventions | Organisational delivery and implementation interventions |
| Types of outcome measures | Short term: under one week to eight weeks Medium term: over eight weeks to 26 weeks Long term: over 26 weeks | |
| Primary outcomes (prevention studies) | Incidence rate  Incidence proportion | |
| Primary outcomes (treatment studies) | Pressure injury healing  Time to complete wound healing | |
| Secondary outcomes | Staff and patient satisfaction, patient quality of life, and adverse events. | |
|  |  | Barriers and facilitators to implementation |
