# Supplementary material for: Effectiveness of Organisational Strategies for Pressure Injury Prevention and Treatment in Acute Hospital Settings: A Systematic Review
Source: J Adv Nurs. 2025 Jun 5;82(3):2004–21. doi: 10.1111/jan.17090 (PMC12907601; doi:10.1111/jan.17090)
Supplement: Supplementary file 2 — Appendix S2. [file JAN-82-2004-s001.docx]

**Appendix B. Search Strategy**

**Database: Ovid MEDLINE**

Ovid MEDLINE(R) ALL <1946 to November 22, 2023>

1 exp Pressure Ulcer/ 14082

2 (pressure adj1 (ulcer* or sore* or injur*)).tw. 13638

3 (decubitus adj1 (ulcer* or sore*)).tw. 1897

4 (bedsore* or "bed sore*").tw. 841

5 1 or 2 or 3 or 4 19740

6 exp Role/ 113826

7 exp Practice Patterns, Physicians'/ 67230

8 exp Practice Patterns, Nurses'/ 2968

9 exp Practice patterns, pharmacists'/ 26

10 ((shift* or chang* or replac* or substitut* or transfer* or delegat* or expand* or extend* or increas* or empower*) adj4 (role* or boundar* or pattern* or professional* or practice* or responsibilit*)).ti,ab. 231763

11 ((nurs* or physician* or practitioner* or pharmacist* or therapist* or assistant* or doctor* or clinician* or podiatrist* or physiotherapist* or nutritionist* or dietician* or "occupational therapist*" or orthotist* or Prosthetist* or profession*) adj4 (role* or skill* or duty or duties or task* or responsibilit* or charg* or reprofil*)).ti,ab. 109317

12 6 or 7 or 8 or 10 or 11 487460

13 exp Patient Care Team/ 73020

14 exp Interprofessional Relations/ 72675

15 (interdisciplinar* or multidisciplinar* or interprofessional* or "multi profession*").ti,ab. 182489

16 13 or 14 or 15 289083

17 exp Patient Care Management/ 926162

18 exp Continuity of Patient Care/ 293581

19 exp Nursing Care/ 141957

20 exp Health Planning/ 375376

21 exp Delivery of Health Care/ 1238307

22 Patient Safety/ 25709

23 pathway*.ti,ab. 1462971

24 ((integrat* or comprehensive or "patient centered" or "patient‐centered" or "patient‐centred" or "patient centred" or continu* or seamless or "re organis*" or "co ordinat*" or coordinat* or transmural*) adj4 (care or system* or service* or deliv*)).ti,ab. 208964

25 ((case or care) adj4 ("co ordination" or coordination or management or meeting* or discussion* or consultat* or refer*)).ti,ab. 119358

26 ((skill or team or staff* or grade) adj4 (mix or substitut* or "re profil*")).ti,ab. 1760

27 (staff* adj4 level*).ti,ab. 6061

28 exp Telemedicine/ 45868

29 (telemedicine or telehealth or telecommunic* or teleconsult*).ti,ab. 35079

30 ((refer* or consult*) adj4 (special* or expert*)).ti,ab. 66261

31 17 or 18 or 19 or 20 or 21 or 22 or 23 or 24 or 25 or 26 or 27 or 28 or 29 or 30 3749746

32 Personal Satisfaction/ 24716

33 exp "Reinforcement (Psychology)"/ 62326

34 exp Motivation/ 196161

35 exp Reward/ 26984

36 "Reimbursement, Incentive"/ 4794

37 ("personal satisfaction" or reinforcement or motivation or reward* or reimbursement* or incentiv*).ti,ab. 244200

38 32 or 33 or 34 or 35 or 36 or 37 451148

39 exp Health Personnel/ 621483

40 38 and 39 33003

41 Job Satisfaction/ 28912

42 Workplace/ 30046

43 Organizational culture/ 19059

44 ((organi?ation* or work*) adj3 (cultur* or climate or ethos)).ti,ab. 13546

45 organi?ational.ti,ab. 74397

46 40 or 41 or 42 or 43 or 44 or 45 168387

47 exp Implementation Science/ 1348

48 Guideline Adherence/ 35150

49 "Patient Acceptance of Health Care"/ 55322

50 "treatment adherence and compliance"/ 1069

51 "Quality of Health Care"/ 77738

52 quality indicators, health care/ 17478

53 exp clinical audit/ 22992

54 Quality assurance, health care/ or Benchmarking/ 74195

55 Clinical Competence/ 106084

56 ((compliance or adhere* or training or implement* or recommend* or disseminat* or according) adj3 guideline*).ti,ab. 86467

57 (adhere* or compli* or disseminat* or accordance or adopt* or barrier* or facilit* or "top down" or "bottom up" or utili?ation).ti,ab. 3656698

58 47 or 48 or 49 or 50 or 51 or 52 or 53 or 54 or 55 or 56 or 57 3976966

59 Interprofessional Education/ 512

60 education, medical, continuing/ or education, nursing, continuing/ or education, pharmacy, continuing/ or education, professional, retraining/ 50591

61 ("education outreach" or "educational material*" or "local consensus process" or "tailor* intervention*" or "local opinion leader*" or "reminder*" or "educational meeting*" or "printed educational material*").ti,ab. 28377

62 (opinion adj3 leader).ti,ab. 253

63 ((physician* or clinician* or doctor* or nurse* or professional*) adj3 leader*).ti,ab. 9243

64 (audit adj1 feedback).ti,ab. 250

65 59 or 60 or 61 or 62 or 63 or 64 88424

66 randomized controlled trial.pt. 603937

67 controlled clinical trial.pt. 95471

68 Randomized Controlled Trials as Topic/ 165492

69 Clinical Trials as Topic/ 201457

70 (random* or trial* or control* or placebo).ti,ab. 6056684

71 non randomized controlled trials as topic/ 1068

72 interrupted time series analysis/ 1945

73 controlled before after studies/ 741

74 ((before adj5 after) or (pre adj5 post) or ((pretest or "pre test") and (posttest or "post test")) or quasiexperiment* or "quasi experiment*" or "pseudo experiment*" or pseudoexperiment* or evaluat* or "time series" or "time point*" or "repeated measur*").ti,ab. 5076879

75 66 or 67 or 68 or 69 or 70 or 71 or 72 or 73 or 74 9778527

76 12 or 16 or 31 or 38 or 46 or 58 or 65 7621376

77 5 and 75 and 76 3318

78 exp animals/ not humans.sh. 5173743

79 77 not 78 3287

80 limit 79 to (yr="2012 - 2024" and english) 1836

**Database: Cochrane Central Register of Controlled Trials search strategy**

ID Search Hits

#1 MeSH descriptor: [Pressure Ulcer] explode all trees 1084

#2 (pressure near/1 (ulcer* or sore* or injur*)) 2690

#3 (decubitus near/1 (ulcer* or sore*)) 210

#4 (bedsore* or bed NEXT sore*) 270

#5 #1 OR #2 OR #3 OR #4 2883

#6 MeSH descriptor: [Role] explode all trees 1761

#7 MeSH descriptor: [Practice Patterns, Physicians'] explode all trees 2039

#8 MeSH descriptor: [Practice Patterns, Nurses'] explode all trees 208

#9 MeSH descriptor: [Practice Patterns, Pharmacists'] explode all trees 2

#10 (((shift* or chang* or replac* or substitut* or transfer* or delegat* or expand* or extend* or increas* or empower*) near/4 (role* or boundar* or pattern* or professional* or practice* or responsibilit*))):ti OR (((shift* or chang* or replac* or substitut* or transfer* or delegat* or expand* or extend* or increas* or empower*) near/4 (role* or boundar* or pattern* or professional* or practice* or responsibilit*))):ab 13567

#11 (((nurs* or physician* or practitioner* or pharmacist* or therapist* or assistant* or doctor* or clinician* or podiatrist* or physiotherapist* or nutritionist* or dietician* or occupational NEXT therapist* or orthotist* or Prosthetist* or profession*) near/4 (role* or skill* or duty or duties or task* or responsibilit* or charg* or reprofil*))):ti OR (((nurs* or physician* or practitioner* or pharmacist* or therapist* or assistant* or doctor* or clinician* or podiatrist* or physiotherapist* or nutritionist* or dietician* or occupational NEXT therapist* or orthotist* or Prosthetist* or profession*) near/4 (role* or skill* or duty or duties or task* or responsibilit* or charg* or reprofil*))):ab 6126

#12 #6 or #7 or #8 or #9 or #10 or #11 22743

#13 MeSH descriptor: [Patient Care Team] explode all trees 2324

#14 MeSH descriptor: [Interprofessional Relations] explode all trees 816

#15 ((interdisciplinar* or multidisciplinar* or interprofessional* or multi NEXT profession*)):ti OR ((interdisciplinar* or multidisciplinar* or interprofessional* or multi NEXT profession*)):ab 12129

#16 #13 or #14 or #15 14060

#17 MeSH descriptor: [Patient Care Management] explode all trees 43173

#18 MeSH descriptor: [Continuity of Patient Care] explode all trees 40035

#19 MeSH descriptor: [Nursing Care] explode all trees 2376

#20 MeSH descriptor: [Health Planning] explode all trees 6917

#21 MeSH descriptor: [Delivery of Health Care] explode all trees 68141

#22 MeSH descriptor: [Patient Safety] explode all trees 1082

#23 (pathway*):ti OR (pathway*):ab 26719

#24 (((integrat* or comprehensive or "patient centered" or "patient‐centered" or "patient‐ centred" or "patient centred" or continu* or seamless or re NEXT organis* or co NEXT ordinat* or coordinat* or transmural*) near/4 (care or system* or service* or deliv*))):ti OR (((integrat* or comprehensive or "patient centered" or "patient‐centered" or "patient‐ centred" or "patient centred" or continu* or seamless or re NEXT organis* or co NEXT ordinat* or coordinat* or transmural*) near/4 (care or system* or service* or deliv*))):ab 18319

#25 (((case or care) near/4 ("co ordination" or coordination or management or meeting* or discussion* or consultat* or refer*))):ti OR (((case or care) near/4 ("co ordination" or coordination or management or meeting* or discussion* or consultat* or refer*))):ab 16181

#26 (((skill or team or staff* or grade) near/4 (mix or substitut* or re NEXT profil*))):ti OR (((skill or team or staff* or grade) near/4 (mix or substitut* or re NEXT profil*))):ab 74

#27 ((staff* near/4 level*)):ti OR ((staff* near/4 level*)):ab 470

#28 MeSH descriptor: [Telemedicine] explode all trees 4989

#29 ((telemedicine or telehealth or telecommunic* or teleconsult*)):ti OR ((telemedicine or telehealth or telecommunic* or teleconsult*)):ab 5743

#30 (((refer* or consult*) near/4 (special* or expert*))):ti OR (((refer* or consult*) near/4 (special* or expert*))):ab 3525

#31 #17 or #18 or #19 or #20 or #21 or #22 or #23 or #24 or #25 or #26 or #27 or #28 or #29 or #30 180485

#32 MeSH descriptor: [Personal Satisfaction] this term only 1522

#33 MeSH descriptor: [Reinforcement, Psychology] explode all trees 3097

#34 MeSH descriptor: [Motivation] explode all trees 12463

#35 MeSH descriptor: [Reward] explode all trees 1497

#36 MeSH descriptor: [Reimbursement, Incentive] this term only 184

#37 (("personal satisfaction" or reinforcement or motivation or reward* or reimbursement* or incentiv*)):ti OR (("personal satisfaction" or reinforcement or motivation or reward* or reimbursement* or incentiv*)):ab 26395

#38 #32 or #33 or #34 or #35 or #36 or #37 37510

#39 MeSH descriptor: [Health Personnel] explode all trees 15720

#40 #38 or #39 52218

#41 MeSH descriptor: [Job Satisfaction] this term only 396

#42 MeSH descriptor: [Workplace] this term only 1315

#43 MeSH descriptor: [Organizational Culture] this term only 148

#44 (((organi?ation* or work*) near/3 (cultur* or climate or ethos))):ti OR (((organi?ation* or work*) near/3 (cultur* or climate or ethos))):ab 446

#45 (organi?ational):ti OR (organi?ational):ab 2936

#46 #40 or #41 or #42 or #43 or #44 or #45 55854

#47 MeSH descriptor: [Implementation Science] explode all trees 106

#48 MeSH descriptor: [Guideline Adherence] this term only 1611

#49 MeSH descriptor: [Patient Acceptance of Health Care] this term only 4329

#50 MeSH descriptor: [Treatment Adherence and Compliance] this term only 201

#51 MeSH descriptor: [Quality of Health Care] this term only 1293

#52 MeSH descriptor: [Quality Indicators, Health Care] this term only 350

#53 MeSH descriptor: [Clinical Audit] explode all trees 424

#54 MeSH descriptor: [Quality Assurance, Health Care] this term only 927

#55 MeSH descriptor: [Benchmarking] this term only 268

#56 MeSH descriptor: [Clinical Competence] this term only 5074

#57 (((compliance or adhere* or training or implement* or recommend* or disseminat* or according) near/3 guideline*)):ti OR (((compliance or adhere* or training or implement* or recommend* or disseminat* or according) near/3 guideline*)):ab 14918

#58 ((adhere* or compli* or disseminat* or accordance or adopt* or barrier* or facilit* or "top down" or "bottom up" or utili?ation)):ti OR ((adhere* or compli* or disseminat* or accordance or adopt* or barrier* or facilit* or "top down" or "bottom up" or utili?ation)):ab 335995

#59 #47 or #48 or #49 or #50 or #51 or #52 or #53 or #54 or #55 or #56 or #57 or #58 353462

#60 MeSH descriptor: [Interprofessional Education] this term only 12

#61 MeSH descriptor: [Education, Medical, Continuing] this term only 894

#62 MeSH descriptor: [Education, Nursing, Continuing] this term only 355

#63 MeSH descriptor: [Education, Pharmacy, Continuing] this term only 39

#64 MeSH descriptor: [Education, Professional, Retraining] this term only 10

#65 (("education outreach" or educational NEXT material* or "local consensus process" or tailor* NEXT intervention* or local NEXT opinion NEXT leader* or reminder* or educational NEXT meeting* or printed NEXT educational NEXT material*)):ti OR (("education outreach" or educational NEXT material* or "local consensus process" or tailor* NEXT intervention* or local NEXT opinion NEXT leader* or reminder* or educational NEXT meeting* or printed NEXT educational NEXT material*)):ab 11433

#66 ((opinion near/3 leader)):ti OR ((opinion near/3 leader)):ab 107

#67 (((physician* or clinician* or doctor* or nurse* or professional*) near/3 leader*)):ti OR (((physician* or clinician* or doctor* or nurse* or professional*) near/3 leader*)):ab 291

#68 ((audit near/1 feedback)):ti OR ((audit near/1 feedback)):ab 121

#69 #60 or #61 or #62 or #63 or #64 or #65 or #66 or #67 or #68 13048

#70 MeSH descriptor: [Randomized Controlled Trial] this term only 37

#71 MeSH descriptor: [Controlled Clinical Trial] this term only 7

#72 MeSH descriptor: [Randomized Controlled Trials as Topic] this term only 52385

#73 MeSH descriptor: [Clinical Trials as Topic] this term only 40781

#74 ((random* or trial* or control* or placebo)):ti OR ((random* or trial* or control* or placebo)):ab 1670021

#75 MeSH descriptor: [Non-Randomized Controlled Trials as Topic] this term only 230

#76 MeSH descriptor: [Interrupted Time Series Analysis] this term only 121

#77 MeSH descriptor: [Controlled Before-After Studies] this term only 145

#78 (((before near/5 after) or (pre near/5 post) or ((pretest or "pre test") or (posttest or "post test")) or quasiexperiment* or quasi NEXT experiment* or pseudo NEXT experiment* or pseudoexperiment* or evaluat* or "time series" or time NEXT point* or repeated NEXT measur*)):ti OR (((before near/5 after) or (pre near/5 post) or ((pretest or "pre test") or (posttest or "post test")) or quasiexperiment* or quasi NEXT experiment* or pseudo NEXT experiment* or pseudoexperiment* or evaluat* or "time series" or time NEXT point* or repeated NEXT measur*)):ab 786939

#79 #70 or #71 or #72 or #73 or #74 or #75 or #76 or #77 or #78 1789306

#80 #12 or #16 or #31 or #38 or #46 or #59 or #69 520408

#81 #5 and #79 and #80 1072

#82 #81 with Cochrane Library publication date Between Jan 2023 and Dec 2023 82

**Database: Ovid Embase**

1.exp Pressure Ulcer/

2.(pressure adj (ulcer* or sore* or injur*)).tw.

3.(decubitus adj (ulcer* or sore*)).tw.

4.(bedsore* or "bed sore*").tw.

5.1 or 2 or 3 or 4

6.exp Role/

7.exp Practice Patterns, Physicians'/

8.exp Practice Patterns, Nurses'/

9.exp Nursing/

10.exp Practice patterns, pharmacists'/

11.((shift* or chang* or replac* or substitut* or transfer* or delegat* or expand* or extend* or increas* or empower*) adj4 (role* or boundar* or pattern* or professional* or practice* or responsibilit*)).ti,ab.

12.((nurs* or physician* or practitioner* or pharmacist* or therapist* or assistant* or doctor* or clinician* or podiatrist* or physiotherapist* or nutritionist* or dietician* or "occupational therapist*" or orthotist* or Prosthetist* or profession*) adj4 (role* or skill* or duty or duties or task* or responsibilit* or charg* or reprofil*)).ti,ab.

13.6 or 7 or 8 or 11 or 12

14.exp Patient Care Team/

15.exp Interprofessional Relations/

16.(interdisciplinar* or multidisciplinar* or interprofessional* or "multi profession*").ti,ab.

17.14 or 15 or 16

18.exp Patient Care Management/

19.exp Continuity of Patient Care/

20.exp Nursing Care/

21.exp Health Planning/

22.exp Delivery of Health Care/

23.Patient Safety/

24.pathway*.ti,ab.

25.((integrat* or comprehensive or "patient centered" or "patient‐centered" or "patient‐centred" or "patient centred" or continu* or seamless or "re organis*" or "co ordinat*" or coordinat* or transmural*) adj4 (care or system* or service* or deliv*)).ti,ab.

26.((case or care) adj4 ("co ordination" or coordination or management or meeting* or discussion* or consultat* or refer*)).ti,ab.

27.((skill or team or staff* or grade) adj4 (mix or substitut* or "re profil*")).ti,ab.

28.(staff* adj4 level*).ti,ab.

29.(telemedicine or telehealth or telecommunic* or teleconsult*).ti,ab.

30.((refer* or consult*) adj4 (special* or expert*)).ti,ab.

31.18 or 19 or 20 or 21 or 22 or 23 or 24 or 25 or 26 or 27 or 28 or 29 or 30

32.Personal Satisfaction/

33.exp "Reinforcement (Psychology)"/

34.exp Motivation/

35.exp Reward/

36."Reimbursement, Incentive"/

37.32 or 33 or 34 or 35 or 36

38.exp Health Personnel/

39.37 and 38

40.Job Satisfaction/

41.Workplace/

42.Organizational culture/

43.((organi?ation* or work*) adj3 (cultur* or climate or ethos)).ti,ab.

44.39 or 40 or 41 or 42 or 43

45.((nurs* or physician* or practitioner* or pharmacist* or therapist* or assistant* or doctor* or clinician* or podiatrist* or physiotherapist* or nutritionist* or dietician* or "occupational therapist*" or orthotist* or Prosthetist* or profession*) adj4 (satisfaction* or incentiv* or reward* or reinforcem* or motivation*)).ti,ab.

46.organi?ational.ti,ab.

47.exp Implementation Science/

48.Guideline Adherence/

49."Patient Acceptance of Health Care"/

50."treatment adherence and compliance"/

51."Quality of Health Care"/

52.quality indicators, health care/

53.exp clinical audit/

54.Quality assurance, health care/ or Benchmarking/

55.Clinical Competence/

56.((compliance or adhere* or training or implement* or recommend* or disseminat* or according) adj3 guideline*).ti,ab.

57.(adhere* or compli* or disseminat* or accordance or adopt* or barrier* or facilit* or "top down" or "bottom up" or utili?ation).ti,ab.

58.Interprofessional Education/

59.education, medical, continuing/ or education, nursing, continuing/ or education, pharmacy, continuing/ or education, professional, retraining/

60.("education outreach" or "educational material*" or "local consensus process" or "tailor* intervention*" or "local opinion leader*" or "reminder*" or "educational meeting*" or "printed educational material*").ti,ab.

61.(opinion adj3 leader).ti,ab.

62.((physician* or clinician* or doctor* or nurse* or professional*) adj3 leader*).ti,ab.

63."audit and feedback".ti,ab.

64.45 or 46 or 47 or 48 or 49 or 50 or 51 or 52 or 53 or 54 or 55 or 56 or 57 or 58 or 59 or 60 or 61 or 62 or 63

65.randomized controlled trial.pt.

66.controlled clinical trial.pt.

67.Randomized Controlled Trials as Topic/

68.Clinical Trials as Topic/

69.(random* or trial* or control* or placebo).ti,ab.

70.non randomized controlled trials as topic/

71.interrupted time series analysis/

72.controlled before after studies/

73.((before adj5 after) or (pre adj5 post) or ((pretest or "pre test") and (posttest or "post test")) or quasiexperiment* or "quasi experiment*" or "pseudo experiment*" or pseudoexperiment* or evaluat* or "time series" or "time point*" or "repeated measur*").ti,ab.

74.exp animals/ not humans.sh.

75.65 or 66 or 67 or 68 or 69 or 70 or 71 or 72 or 73

76.13 or 17 or 31 or 37 or 44 or 64

77.5 and 75 and 76

78.77 not 74

**Database: EBSCO CINAHL Complete**

S1 (MH "Pressure Ulcer+")

S2 (pressure N1 (ulcer* or sore* or injur*))

S3 (decubitus N1 (ulcer* or sore*))

S4 bedsore* or bed sore*

S5 S1 OR S2 OR S3 OR S4

S6 (MH "Role+") OR (MH "Practice Patterns") OR (MH "Nursing Practice+")

S7 TI ( ((shift* or chang* or replac* or substitut* or transfer* or delegat* or

expand* or extend* or increas* or empower*) N4 (role* or boundar* or

pattern* or professional* or practice* or responsibilit*)) ) OR AB ( ((shift* or

chang* or replac* or substitut* or transfer* or delegat* or expand* or extend*

or increas* or empower*) N4 (role* or boundar* or pattern* or professional* or

practice* or responsibilit*)) )

S8 TI ( ((nurs* or physician* or practitioner* or pharmacist* or therapist* or

assistant* or doctor* or clinician* or podiatrist* or physiotherapist* or

nutritionist* or dietician* or "occupational therapist*" or orthotist* or

Prosthetist* or profession*) N4 (role* or skill* or duty or duties or task* or

responsibilit* or charg* or reprofil*)) ) OR AB ( ((nurs* or physician* or

practitioner* or pharmacist* or therapist* or assistant* or doctor* or clinician*

or podiatrist* or physiotherapist* or nutritionist* or dietician* or "occupational

therapist*" or orthotist* or Prosthetist* or profession*) N4 (role* or skill* or duty

or duties or task* or responsibilit* or charg* or reprofil*)) )

S9 S6 OR S7 OR S8

S10 (MH "Multidisciplinary Care Team") OR (MH "Interprofessional Relations+")

S11 TI ( (interdisciplinar* or multidisciplinar* or interprofessional* or "multi

profession*") ) OR AB ( (interdisciplinar* or multidisciplinar* or

interprofessional* or "multi profession*") )

S12 S10 OR S11

S13 (MH "Patient Centered Care") OR (MH "Continuity of Patient Care+") OR (MH

"Nursing Care") OR (MH "Health and Welfare Planning+") OR (MH "Health

Care Delivery+") OR (MH "Patient Safety") OR (MH "Health Care Errors") OR

(MH "Sentinel Event") OR (MH "Patient Care+")

S14 TI pathway* OR AB pathway*

S15 TI ( ((integrat* or comprehensive or "patient centered" or "patient‐centered" or

"patient‐centred" or "patient centred" or continu* or seamless or "re organis*"

or "co ordinat*" or coordinat* or transmural*) N4 (care or system* or service*

or deliv*)) ) OR AB ( ((integrat* or comprehensive or "patient centered" or

"patient‐centered" or "patient‐centred" or "patient centred" or continu* or

seamless or "re organis*" or "co ordinat*" or coordinat* or transmural*) N4

(care or system* or service* or deliv*)) )

S16 TI ( ((case or care) N4 ("co ordination" or coordination or management or

meeting* or discussion* or consultat* or refer*)) ) OR AB ( ((case or care) N4

("co ordination" or coordination or management or meeting* or discussion* or

consultat* or refer*)) )

S17 TI ( ((skill or team or staff* or grade) N4 (mix or substitut* or "re profil*")) ) OR

AB ( ((skill or team or staff* or grade) N4 (mix or substitut* or "re profil*")) )

S18 TI (staff* N4 level*) OR AB (staff* N4 level*)

S19 TI ( (telemedicine or telehealth or telecommunic* or teleconsult*) ) OR AB (

(telemedicine or telehealth or telecommunic* or teleconsult*) )

S20 TI ( ((refer* or consult*) N4 (special* or expert*)) ) OR AB ( ((refer* or

consult*) N4 (special* or expert*)) )

S21 (MH "Telehealth+") OR (MH "Telemedicine+") OR (MH "Telenursing") OR (MH

"Telenutrition") OR (MH "Telecommunications+")

S22 S13 OR S14 OR S15 OR S16 OR S17 OR S18 OR S19 OR S20 OR S21

S23 (MH "Personal Satisfaction+") OR (MH "Reinforcement (Psychology)+") OR

(MH "Motivation+") OR (MH "Reward") OR (MH "Reimbursement, Incentive")

OR TI ("personal satisfaction" or reinforcement or motivation or reward* or

reimbursement* or incentiv*) OR AB ("personal satisfaction" or reinforcement

or motivation or reward* or reimbursement* or incentiv*)

S24 (MH "Health Personnel+")

S25 S23 AND S24

S26 (MH "Job Satisfaction+") OR (MH "Work Environment+") OR (MH

"Organizational Culture+") OR (MH "Employee Incentive Programs") OR (MH

"Physician Incentive Plans")

S27 TI organi?ational OR AB organi?ational

S28 S25 OR S26 OR S27

S29 (MH "Implementation Science") OR (MH "Guideline Adherence") OR (MH

"Quality of Health Care") OR (MH "Professional Compliance") OR (MH

"Patient Compliance") OR (MH "Nursing Audit") OR (MH "Audit") OR (MH

"Clinical Indicators") OR (MH "Benchmarking") OR (MH "Quality Assurance")

OR (MH "Clinical Competence")

S30 TI ( ((compliance or adhere* or training or implement* or recommend* or

disseminat* or according) N3 guideline*) ) OR AB ( ((compliance or adhere*

or training or implement* or recommend* or disseminat* or according) N3

guideline*) )

S31 TI ( (adhere* or compli* or disseminat* or accordance or adopt* or barrier* or

facilit* or "top down" or "bottom up" or utili?ation) ) OR AB ( (adhere* or

compli* or disseminat* or accordance or adopt* or barrier* or facilit* or "top

down" or "bottom up" or utili?ation) )

S32 (MH "Education, Interdisciplinary")

S33 TI ( ((organi?ation* or work*) N3 (cultur* or climate or ethos)) ) OR AB (

((organi?ation* or work*) N3 (cultur* or climate or ethos)) )

S34 (MH "Education, Continuing+") OR (MH "Refresher Courses")

S35 TI ( ("education outreach" or "educational material*" or "local consensus

process" or "tailor* intervention*" or "local opinion leader*" or "reminder*" or

"educational meeting*" or "printed educational material*") ) OR AB (

("education outreach" or "educational material*" or "local consensus process"

or "tailor* intervention*" or "local opinion leader*" or "reminder*" or

"educational meeting*" or "printed educational material*") )

S36 TI (opinion N3 leader) OR AB (opinion N3 leader)

S37 TI ( ((physician* or clinician* or doctor* or nurse* or professional*) N3 leader*)

) OR AB ( ((physician* or clinician* or doctor* or nurse* or professional*) N3

leader*) )

S38 TI ( audit N1 feedback ) OR AB ( audit N1 feedback )

S39 S28 OR S29 OR S30 OR S31 OR S32 OR S33 OR S34 OR S35 OR S36 OR

S37 OR S38

S40 (MH "Clinical Trials+") OR (MH "Randomized Controlled Trials+") OR (MH

"Random Assignment")

S41 PT controlled clinical trial or randomized controlled trial or clinical trial

S42 TI ( random* or trial* or control* or placebo ) OR AB ( random* or trial* or

control* or placebo )

S43 (MH "Interrupted Time Series Analysis") OR (MH "Controlled Before-After

Studies")

S44 TI ( ((before N5 after) or (pre N5 post) or ((pretest or "pre test") and (posttest

or "post test")) or quasiexperiment* or "quasi experiment*" or "pseudo

experiment*" or pseudoexperiment* or evaluat* or "time series" or "time

point*" or "repeated measur*") ) OR AB ( ((before N5 after) or (pre N5 post) or

((pretest or "pre test") and (posttest or "post test")) or quasiexperiment* or

"quasi experiment*" or "pseudo experiment*" or pseudoexperiment* or

evaluat* or "time series" or "time point*" or "repeated measur*") )

S45 S40 OR S41 OR S42 OR S43 OR S44

S46 S9 OR S12 OR S22 OR S39

S47 S5 AND S45 AND S46

**Database: Web of Science Core Collection**

#1 (pressure NEAR/1 (ulcer* or sore* or injur*)) (Topic)

#2 (decubitus NEAR/1 (ulcer* or sore*)) (Topic)

#3 bedsore* or "bed sore*" (Topic)

#4 #1 or #2 or #3

#5 ((shift* or chang* or replac* or substitut* or transfer* or delegat* or expand* or extend* or increas* or empower*) NEAR/4 (role* or boundar* or pattern* or professional* or practice* or responsibilit*)) (Title) OR ((shift* or chang* or replac* or substitut* or transfer* or delegat* or expand* or extend* or increas* or empower*) NEAR/4 (role* or boundar* or pattern* or professional* or practice* or responsibilit*)) (Abstract)

#6 ((nurs* or physician* or practitioner* or pharmacist* or therapist* or assistant* or doctor* or clinician* or podiatrist* or physiotherapist* or nutritionist* or dietician* or "occupational therapist*" or orthotist* or Prosthetist* or profession*) NEAR/4 (role* or skill* or duty or duties or task* or responsibilit* or charg* or reprofil*)) (Title) OR ((nurs* or physician* or practitioner* or pharmacist* or therapist* or assistant* or doctor* or clinician* or podiatrist* or physiotherapist* or nutritionist* or dietician* or "occupational therapist*" or orthotist* or Prosthetist* or profession*) NEAR/4 (role* or skill* or duty or duties or task* or responsibilit* or charg* or reprofil*)) (Abstract)

#7 #5 OR #6

#8 (interdisciplinar* or multidisciplinar* or interprofessional* or "multi profession*") (Title) OR (interdisciplinar* or multidisciplinar* or interprofessional* or "multi profession*")

#9 pathway* (Title) OR pathway* (Abstract)

#10 ((integrat* or comprehensive or “patient centered” or “patient-centered” or “patient-centred” or “patient centred” or continu* or seamless or “re organis*” or “co ordinat*” or coordinat* or transmural*) NEAR/4 (care or system* or service* or deliv*)) (Title) OR ((integrat* or comprehensive or “patient centered” or “patient-centered” or “patient-centred” or “patient centred” or continu* or seamless or “re organis*” or “co ordinat*” or coordinat* or transmural*) NEAR/4 (care or system* or service* or deliv*)) (Abstract)

#11 ((case or care) NEAR/4 ("co ordination" or coordination or management or meeting* or discussion* or consultat* or refer*)) (Title) OR ((case or care) NEAR/4 ("co ordination" or coordination or management or meeting* or discussion* or consultat* or refer*))

#12 ((skill or team or staff* or grade) NEAR/4 (mix or substitut* or "re profil*")) (Title) OR ((skill or team or staff* or grade) NEAR/4 (mix or substitut* or "re profil*")) (Abstract)

#13 (staff* NEAR/4 level*) (Title) OR (staff* NEAR/4 level*) (Abstract)

#14 (telemedicine or telehealth or telecommunic*) (Title) OR (telemedicine or telehealth or telecommunic*) (Abstract)

#15 ((refer* or consult*) NEAR/4 (special* or expert*)) (Title) OR ((refer* or consult*) NEAR/4 (special* or expert*)) (Abstract)

#16 #8 or #9 or #10 or #11 or #12 or #13 or #14 or #15

#17 ("personal satisfaction" or reinforcement or motivation or reward* or reimbursement* or incentiv*) (Title) OR ("personal satisfaction" or reinforcement or motivation or reward* or reimbursement* or incentiv*) (Abstract)

#18 "health personnel" (Title) OR "health personnel" (Abstract)

#19 #17 and #18

#20 (organi?ation* or work*) NEAR/3 (cultur* or climate or ethos) (Title) OR (organi?ation* or work*) NEAR/3 (cultur* or climate or ethos) (Abstract)

#21 organi?ational (Title) OR organi?ational (Abstract)

#22 #20 or #21

#23 ((compliance or adhere* or training or implement* or recommend* or disseminat* or according) NEAR/3 guideline*) (Title) OR ((compliance or adhere* or training or implement* or recommend* or disseminat* or according) NEAR/3 guideline*) (Abstract)

#24 (adhere* or compli* or disseminat* or accordance or adopt* or barrier* or facilit* or "top down" or "bottom up" or utili?ation) (Title) OR (adhere* or compli* or disseminat* or accordance or adopt* or barrier* or facilit* or "top down" or "bottom up" or utili?ation) (Abstract)

#25 #23 or #24

#26 ("education outreach" or "educational material*" or "local consensus process" or "tailor* intervention*" or "local opinion leader*" or reminder* or "educational meeting*" or "printed educational material*") (Title) OR ("education outreach" or "educational material*" or "local consensus process" or "tailor* intervention*" or "local opinion leader*" or reminder* or "educational meeting*" or "printed educational material*") (Abstract)

#27 (opinion NEAR/3 leader) (Title) OR (opinion NEAR/3 leader) (Abstract)

#28 ((physician* or clinician* or doctor* or nurse* or professional*) NEAR/3 leader*) (Title) OR ((physician* or clinician* or doctor* or nurse* or professional*) NEAR/3 leader*) (Abstract)

#29 (audit NEAR/1 feedback) (Title) OR (audit NEAR/1 feedback) (Abstract)

#30 #26 or #27 or #28 or #29

#31 #7 or #16 or #19 or #22 or #28

#32 #31 and #4

#33 (random* or placebo* or trial*) (Title) OR (random* or placebo* or trial*) (Abstract)

#34 ((before NEAR/5 after) or (pre NEAR/5 post) or ((pretest or pre test) or (posttest or post test)) or quasiexperiment* or "quasi experiment*" or pseudoexperiment* or "pseudo experiment*" or evaluat* or "time series" or "time point*" or "repeated measur*") (Title) OR ((before NEAR/5 after) or (pre NEAR/5 post) or ((pretest or pre test) and (posttest or post test)) or quasiexperiment* or "quasi experiment*" or pseudoexperiment* or "pseudo experiment*" or evaluat* or "time series" or "time point*" or "repeated measur*") (Abstract)

#35 #33 or #34

#36 #32 and #35

#37 Timespan: 2012-01-01 to 2023-12-31 and English (languages)
